# How to Start with a Clean Crop: Biopesticide Dips Reduce Populations of *Bemisia tabaci* (Hemiptera: Aleyrodidae) on Greenhouse Poinsettia Propagative Cuttings

**DOI:** 10.3390/insects7040048

**Published:** 2016-09-26

**Authors:** Rosemarije Buitenhuis, Michael Brownbridge, Angela Brommit, Taro Saito, Graeme Murphy

**Affiliations:** 1 Vineland Research and Innovation Centre, Vineland Station, Lincoln, ON L0R 2E0, Canada; Michael.Brownbridge@vinelandresearch.com (M.B.); Angie.Crag@gmail.com (A.B.); Taro.Saito@vinelandresearch.com (T.S.); 2 BioLogical Solutions, Welland, ON L3C 2Y3, Canada; graeme.murphy307@gmail.com

**Keywords:** greenhouse, integrated pest management, biopesticides, *Bemisia* whitefly, poinsettia cuttings

## Abstract

(1) Global movement of propagative plant material is a major pathway for introduction of *Bemisia tabaci* (Hemiptera: Aleyrodidae) into poinsettia greenhouses. Starting a poinsettia crop with high pest numbers disrupts otherwise successful biological control programs and widespread resistance of *B. tabaci* against pesticides is limiting growers’ options to control this pest; (2) This study investigated the use of several biopesticides (mineral oil, insecticidal soap, *Beauveria bassiana*, *Isaria fumosorosea*, *Steinernema feltiae*) and combinations of these products as immersion treatments (cutting dips) to control *B. tabaci* on poinsettia cuttings. In addition, phytotoxicity risks of these treatments on poinsettia cuttings, and effects of treatment residues on mortality of commercial whitefly parasitoids (*Eretmocerus eremicus* and *Encarsia formosa*) were determined; (3) Mineral oil (0.1% v/v) and insecticidal soap (0.5%) + *B. bassiana* (1.25 g/L) were the most effective treatments; only 31% and 29%, respectively, of the treated *B. tabaci* survived on infested poinsettia cuttings and *B. tabaci* populations were lowest in these treatments after eight weeks. Phytotoxicity risks of these treatments were acceptable, and dip residues had little effect on survival of either parasitoid, and are considered highly compatible; (4) Use of poinsettia cutting dips will allow growers to knock-down *B. tabaci* populations to a point where they can be managed successfully thereafter with existing biocontrol strategies.

## 1. Introduction

*Bemisia tabaci* (Gennadius) (Hemiptera: Aleyrodidae), also called tobacco, sweet potato or silverleaf whitefly, is an invasive, polyphagous pest worldwide. In greenhouse ornamental production in North America, *B. tabaci* is primarily a pest of poinsettia (*Euphorbia pulcherrima* Willd. ex Klotsch) and is difficult to control, especially if heavy infestations occur at the start of the production cycle [1]. Because greenhouse floriculture relies on the production of an aesthetically perfect crop for retail sale, the visible presence of whiteflies and exuviae negatively impact consumer purchases and, in the case of plants which are exported, may result in rejection of the crop at the border or at the point of delivery which has significant economic implications for the grower. At high infestation levels, feeding by whiteflies causes a reduction in plant vigour and their honeydew promotes the growth of black sooty mould. Although in other crops, *B. tabaci* may be an important vector of plant viruses, in poinsettia this is not a significant issue.

*Bemisia tabaci* is actually a complex of morphologically indistinguishable species [2]. The most prevalent species in North America (formerly considered biotypes) are characterized by a high invasive ability (Middle East-Asia Minor 1 (MEAM1) or B biotype) and an innate ability to rapidly develop a high-level of insecticide resistance that persists in the absence of exposure (Mediterranean or Q biotype) [3,4]. Resistance to conventional pesticides including newer chemistries is observed [5]. In countries such as Canada, where access to registered pesticides is limited, pest resistance and inconsistent control of *B. tabaci* in poinsettia provided by synthetic pesticides prompted a shift to preventative rather than reactive pest management.

Management of *B. tabaci* in poinsettia in Canada has been based on the use of parasitoids (*Eretmocerus mundus* Mercet, *Eretmocerus eremicus* (Rose and Zolnerowich) and/or *Encarsia formosa* Gahan (Hymenoptera: Aphelinidae)), and predators such as *Amblyseius swirskii* Athias-Henriot (Acari: Phytoseiidae) and *Delphastus catalinae* (Horn) (Coleoptera: Coccinellidae). Many growers have used biological control techniques to provide reliable season-long control with minimal to no pesticide inputs [6], but more recent experience in Ontario suggests that biocontrol programs are not as effective as they have been in the past [7]. This situation has been exacerbated by the recent discontinuation of *E. mundus* by commercial insectaries. 

Global movement of propagative plant material (e.g., unrooted cuttings taken from stock plants) is a major pathway for introduction of *B. tabaci* into poinsettia greenhouses. Production of poinsettia cuttings at propagators in the United States, Europe and Central America is characterized by a heavy reliance on chemical pesticides. In spite of these activities, poinsettia cuttings will typically arrive at the grower infested—some heavily—with *B. tabaci*. Studies from the UK, where *B. tabaci* is subject to eradication, demonstrated that it has been intercepted annually in the UK on imported plant material since 1987, in particular on poinsettias [8]. In Ontario (Canada), where *B. tabaci* is not known to overwinter outdoors, surveys of commercial greenhouse operations routinely find this whitefly in young poinsettia plants [4]. Starting a crop with high pest infestations makes it very difficult for growers to successfully use biological controls, which are best used in a preventative rather than a curative manner. The pesticide residues on poinsettia cuttings are also suspected to cause failure of biocontrol programs, as many products are incompatible with beneficial insects through direct toxicity or sub-lethal effects [9]. Furthermore, overreliance on pesticides that are constantly being used at high rates at the propagation facilities means that the whiteflies have probably been repeatedly exposed to any actives registered in Canada, and the effectiveness of any insecticides will be compromised due to the development of resistance. To ensure greater sustainability in poinsettia production, new methods of whitefly control are required that can be applied to cuttings immediately prior to planting to prevent resident whitefly populations developing beyond the control ‘capacity’ of the parasitoids used, and to ensure that effective biological control systems can be maintained through the crop production cycle.

When poinsettia cuttings are received at a greenhouse, they are typically stuck in strips of growing medium and placed under misting to develop roots. At this stage, it is not feasible to apply pesticides because the foliage is constantly rinsed by the frequent mist cycle that is needed to prevent plants from drying. In addition, good coverage to target pests on the underside of the foliage is very difficult to achieve, and systemic insecticides are not taken up by the plants due to the lack of a root system [10]. Potential technologies to suppress pest populations on propagative plant material or cut flowers include non-chemical options such as heat treatments [11,12], irradiation [13] and controlled atmosphere [14,15,16]. Chemical options to mitigate *B. tabaci* such as immersion (dipping) of poinsettia propagative cuttings in conventional pesticides have proven effective [17,18], but worker exposure to the insecticides and waste disposal remain problematic, as well as pest resistance and the potential negative effects of pesticide residues on biocontrol agents. Therefore, research has re-focused on immersion treatments using reduced risk products and biopesticides. *Bemisia tabaci* is susceptible to products based on insecticidal soap or horticultural oil [17,18,19], entomopathogenic fungi such as *Beauveria bassiana* (Balsamo) Vuillemin and *Isaria fumosorosea* Wize [19,20,21], and entomopathogenic nematodes [22].

Earlier research using leaf dips suggested that *B. tabaci* (cryptic species unknown) can be controlled by immersing poinsettia cuttings in reduced risk products and biopesticides such as insecticidal soap, petroleum oil or *B. bassiana*, causing high (up to 100%) mortality of first and second instar *B. tabaci*; egg mortality was generally lower [11,23]. These trials were limited in scope and restricted to a laboratory assessment of efficacy on excised leaves. Synergistic interactions have often been observed when several fungal pathogens have been combined with each other or co-applied with pesticides [24]. Preliminary observations indicated that combining reduced risk products and biopesticides increased treatment efficacy against *B. tabaci* [25]. The current research evaluated biopesticides individually and in combination to see if they are sufficiently effective to be used as immersion treatments in poinsettia production. In addition, whole cuttings were dipped and grown according to commercial practices for several weeks. The phytotoxicity of different dip treatments was also evaluated, and the compatibility of the dip residues on the cuttings with whitefly natural enemies was determined. Ultimately, the dipping technique is intended to suppress pest populations to low levels that then allow parasitoids or predators to be used to prevent further population growth thereafter. This will allow whiteflies to be managed in a sustainable and cost-effective manner, using methods that can be readily implemented in commercial greenhouses.

## 2. Materials and Methods

### 2.1. Plants, Insects and Products

Poinsettia plants (var. Prestige Red) were obtained from a commercial greenhouse (St. Catharines, ON, Canada) in December 2012, pruned back and grown as stock plants in a research greenhouse to provide cuttings that were guaranteed to be free from pesticide residues for the experiments. For the phytotoxicity and compatibility experiments, poinsettia cuttings were received from a commercial poinsettia propagator (Ecke Ranch (now part of Dümmen), Encinitas, CA, USA).

*Bemisia tabaci* used to establish a lab colony were collected from infested poinsettia plants at commercial greenhouses (St. Catharines, ON, Canada). Populations in Canadian greenhouses usually are a mix of MEAM1 and Mediterranean species [4]. The colony was maintained on poinsettia plants in cages inside a growth chamber set at 25 °C, 75% RH, 14:10 L:D for several months and most likely consisted of MEAM1 as it displaces the Mediterranean species without exposure to pesticides or pesticide residues [4].

Products used were: mineral oil (SuffOil-X^®^, Refined mineral oil 80%, BioWorks Inc., Victor, NY, USA), insecticidal soap (Bug B Gon^®^, Potassium salts of fatty acids 47%, Scotts EcoSense^®^; Scotts Canada Ltd., Mississauga, ON, Canada), *Beauveria bassiana* (BotaniGard^®^ 22WP, BioWorks Inc.), *Isaria fumosorosea* (NoFly WP^®^, Natural Industries (now part of Novozymes Corp.), Houston, TX, USA) and *Steinernema feltiae* (Nemasys^®^, BASF Corporation, Research Triangle, NC, USA). Parasitoids, *E. eremicus* and *E. formosa*, were ordered from Biobest Canada (Leamington, ON, Canada).

### 2.2. Phytotoxicity

Four commercial varieties of poinsettia were obtained from a commercial poinsettia propagator: Prestige Red, Enduring Pink, Enduring Marble and Marblestar. Treatments were: control (water), mineral oil (0.5, 0.25 and 0.1% v/v) with and without a water rinse, insecticidal soap (2.0 and 0.5% v/v) and *B. bassiana* (1.25 g/L). Ten cuttings per treatment and per variety were completely immersed in a 1 L beaker containing the treatment solution. The ends of the cuttings stems were then coated in rooting hormone (Stim-Root^®^ # 2, Master Plant Prod Inc., Brampton, ON, Canada) and planted in Oasis Wedge strips (OASIS^®^ Grower Solutions, Smithers-Oasis North America, Kent, OH, USA). All cuttings were placed in a misting house at 25 °C, 100% RH and misted for 5 s every 10 min. After one and two weeks, cuttings were visually inspected one at a time for symptoms of phytotoxicity, including yellowing leaves, burnt leaves, burnt growth tips and lost leaves. Phytotoxicity was quantified as the number of affected leaves. The experiment was laid out as a randomized complete block design with a block of 10 replicates (cuttings) repeated three times over time for a total of 30 replicates per treatment.

A mixed Poisson model was used to study the effect of treatment, variety, week and interactions for fixed effects on total damaged leaves. The GLIMMIX procedure of the SAS program (version 9.2, SAS Institute Inc., Cary, NC, USA) was used with treatment, variety and week as fixed factors, and block as a random factor. Pairwise comparisons were made using the protected Fisher least significant difference (LSD, α = 0.05) test. Results were used to define non-phytotoxic concentrations for the products in the efficacy trials; both mineral oil and insecticidal soap were phytotoxic at the highest rates tested, so these treatments were not included in the efficacy studies.

### 2.3. Compatibility of Entomopathogens with Products in Combination Treatments

Before proceeding to the efficacy trial, the compatibility of selected products with the microbial biopesticides, i.e., effect on viability, was confirmed. Effects of AquaGro (wetting agent often used in commercial practice) (Aquatrols, Paulsboro, NJ, USA), mineral oil and insecticidal soap on the viability of *B. bassiana* conidia and *I. fumosorosea* blastospores were assessed by mixing 0.5 g of fungal product with 5 mL R/O water (control), 1.0% insecticidal soap, 0.02% AquaGro, or 0.1% mineral oil solutions. The prepared suspensions were stored at 4 °C. At t = 0, 1, 2, and 4 h, samples taken from the suspensions were diluted to 10^−3^ and 10^−4^ for plating on quarter-strength Sabouraud Dextrose Agar (EMD Millipore Corporation, Billerica, MA, USA). Two replicate plates were inoculated per dilution and incubated at 25 ± 1 °C, 20 h for *I. fumosorosea* and 16 h for *B. bassiana*. Three 22 mm × 22 mm glass coverslips were then overlain, at random, onto the media. One hundred conidia/blastospores per coverslip were counted under a phase-contrast microscope at 400× magnification and conidial/blastospore viability determined (% germination = (the number of germinated conidia/total of 300 conidia) × 100).

For *S. feltiae*, a small volume of product was suspended in 0.02% AquaGro solution and stored at 4 °C. A total of 100 nematodes were counted under a stereo microscope at t = 0, 1, 2 h to determine viability (% viability = (the number of live nematodes/total of 100 nematodes) × 100).

### 2.4. Efficacy of Reduced Risk Materials

Poinsettia cuttings were treated by immersion in a solution/suspension of the reduced-risk products. Products were applied as individual treatments: (1) control (water); (2) mineral oil 0.25% v/v; (3) mineral oil 0.1% v/v; (4) insecticidal soap 0.5% v/v; (5) *B. bassiana* 1.25 g/L + AquaGro 0.02% v/v; (6) *I. fumosorosea* 3 g/L + AquaGro 0.02% *v*/*v*; (7) *S. feltiae* 2.5 million/L + AquaGro 0.02% v/v; or as combination treatments: (8) mineral oil 0.1% + *B. bassiana* 1.25 g/L; (9) mineral oil 0.1% + *I. fumosorosea* 3 g/L; (10) insecticidal soap 0.5% *v*/*v* + *B. bassiana* 1.25g/L; (11) insecticidal soap 0.5% + *I. fumosorosea* 3 g/L and (12) *B. bassiana* 1.25 g/L + *I. fumosorosea* 3 g/L + AquaGro 0.02% v/v. The experiment was laid out as a randomized complete block design; each block was comprised of 10 replicates (cuttings), and the experiment was repeated twice over time for a total of 20 replicates per treatment. Experiments were conducted from April to May 2013.

Poinsettia cuttings infested with mixed-age immature stages (eggs, first and second instar nymphs) were obtained by placing stock plants into individual cages and introducing 60 adult *B. tabaci* from the colony (sex ratio unknown); plants were held at 25 °C and adults were left on the plants for 12 days. The lower leaves of each plant were removed prior to placement in the cages to encourage oviposition on the shoots that were to be used as cuttings in the experiment. Plants were held for an additional two days before cuttings were taken. Cuttings were wrapped lightly in moist paper towels and held in a cooler at 10 °C for 24 h before being examined under a stereo microscope at 15× to determine the number and stage of *B. tabaci* per leaf. Cuttings with at least 15 *B. tabaci* nymphs and eggs were randomized among treatments and dipped individually for 1–3 s in the assigned treatment, submerging all leaves and most of the stems. The end of the stem was coated with rooting hormone and the cutting was stuck into an Oasis Wedge strip, five cuttings per strip. Stuck cuttings were placed in a greenhouse under misting (25 °C, 100% RH, 5 s every 10 min). Treatments were separated by 30 cm high barriers to prevent cross contamination of whiteflies. After 14 days, cuttings were again examined under the microscope to count the number of dead and live *B. tabaci* eggs, nymphs, pupae and adults. Symptoms of dead *B. tabaci* considered during the post-treatment counts were: desiccation (or detached from leaf or curled up) (common in mineral oil and insecticidal soap treatments), discoloration (common in fungal treatments; especially the red hue in *B. bassiana* treatments), and mycosis (sporulating cadavers in fungal treatments). Percent survival was calculated as (total number of alive larvae, pupae and adults two-weeks post-treatment/total number of eggs and larvae pre-treatment) × 100. Pre-treatment counts were adjusted in case a leaf was lost to ensure the percent survival reflected the result of the treatments. If present, fresh eggs that were laid after dipping were not included, as these would not have been exposed to the dip treatment.

After counting, the cuttings of the control and most efficacious treatments (mineral oil 0.1%, insecticidal soap + *I. fumosorosea* and insecticidal soap + *B. bassiana*) were returned to the misting greenhouse. When cuttings had developed roots, they were potted in 15.3 cm diameter pots, placed individually in cages in a greenhouse compartment at 25 °C and grown according to commercial practices. Two to three weeks after transplanting, plants were pinched above the eighth node according to commercial practice, and pinched material was removed from the cages. The number of immature *B. tabaci* on the plants was determined 8 weeks after the dip treatment to determine if there were any suppressive effects of the treatments on whitefly population development. The number of lateral shoots per plant was also recorded to see if the treatments affected the growth of the poinsettia. In total, 2 blocks of 5 replicates per treatment (total of 10 replicates) were done.

A mixed binomial model was used to study the effect of treatment on the percent whitefly survival. The GLIMMIX procedure of the SAS program was used with treatment as a fixed factor and block as a random factor. Heterogeneity due to treatment was modeled via the group option in the random statement. The Kenward-Roger method was used to calculate the degrees of freedom. Pairwise comparisons were made using the protected Fisher least significant difference (LSD, α = 0.05) test.

A two-way analysis of variance model with repeated measures was used to study the effect of treatment on the further development of whitefly populations. Time was the repeated factor. The MIXED procedure of the SAS program was used with a repeated statement and the covariance structure that minimizes the Akaike criterion. The square root transformation was used so that the normality and the homogeneity of the variance assumptions were met. Pairwise comparisons were made using the protected Fisher least significant difference (LSD, α = 0.05) test.

### 2.5. Compatibility of Dip Treatments with Parasitoids

The effect of mineral oil 0.1% and insecticidal soap + *B. bassiana* on the parasitic wasps *E. eremicus* and *E. formosa* was determined in leaf assays and compared to a pesticide control (abamectin) in July 2013. Parasitoids were received on cards carrying 50 parasitized pupae/card. Cards were placed in clear plastic cups (9 oz, Conex Classic^®^ Clear PET Cups, Dart Canada, Toronto, ON, Canada) containing a cotton swab saturated with sugar water (40% sugar) in a microcentrifuge tube (2 mL) as a food source for emerged adults. Cups were placed in a growth chamber (26 °C, 70% RH) until adult parasitoids emerged. Cuttings (var Prestige Red) were obtained from a commercial poinsettia propagator, transplanted into Oasis strips and placed in a misting greenhouse. Preliminary tests determined that, after one week in the misting greenhouse, there was no significant or obvious detrimental effect on either parasitoid species of pesticide residues which may have been applied at the propagator.

The cuttings were dipped in the following: (1) water (control); (2) mineral oil 0.1%; (3) insecticidal soap 0.5% + *B. bassiana* 1.25 g/L or (4) abamectin 0.3 mL/L (Avid^®^ EC, Syngenta, Guelph, ON, Canada) one week before introduction of parasitoids or (5) abamectin 0.3 mL/L 24 h before introduction of parasitoids. Abamectin residues are known to have a negative effect on *E. eremicus* and *E. formosa* [26] so the treatment was included to verify the effectiveness of the bioassay. For each experimental unit, the first fully formed leaf from the top of the treated cutting was picked and the petiole was put in a microcentrifuge tube (2 mL) containing saturated cotton wool as a source of water. The leaf was placed in a clear plastic cup (9 oz)) with 2 ventilation holes (2.5 cm in diameter on opposite sides of the cup, covered in mesh) and closed with a lid. One week (treatment 1–4) or 24 h (treatment 5) after treating the leaves, twenty adult parasitoids were introduced onto each leaf and 40% sugar water on a cotton swab was provided as a food source. Cups were placed in a growth chamber at 21 °C and 70% RH. After 6 days, the numbers of dead, live and drowned (in sugar water) parasitoids were assessed. Percent survival was calculated as (number of parastioids alive after 6 days/(initial number of parasitoids – drowned parasitoids)) × 100. Each treatment was replicated 23–27 times. Toxicity of treatments was classified according to the International Organization for Biological Control (IOBC) standards for pesticide compatibility [27]. Depending on the survival of beneficial organisms in laboratory tests, the IOBC classifies pesticides into the following four categories: (1) harmless (up to 30% mortality); (2) slightly harmful (30%–79% mortality); (3) moderately harmful (80%–98% mortality); and (4) harmful (99% mortality).

A mixed binomial model was used to study the effect of treatment, species and the interaction of the fixed effects on the survival of the parasitoids. The GLIMMIX procedure of the SAS program was used with treatment and species as fixed factors and block as a random factor. Heterogeneity due to treatment and species was modeled via the group option in the random statement. The method of Kenward-Roger was used to calculate the degrees of freedom. Pairwise comparisons were made using the protected Fisher least significant difference (LSD, α = 0.05) test.

## 3. Results

### 3.1. Phytotoxicity

**Figure 1:**
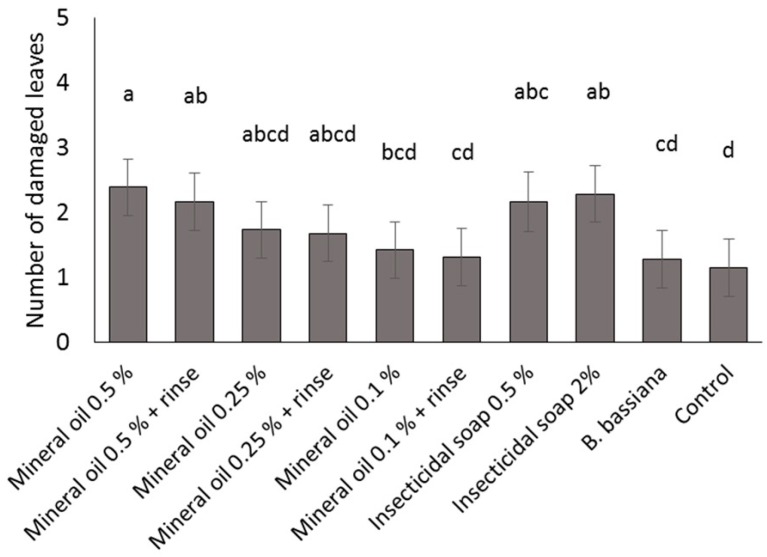
Phytotoxicity (mean ± SE of damaged leaves per cutting) of reduced risk and biopesticide products applied as cutting dips to greenhouse poinsettia. Different letters indicate significant differences among products (α = 0.05).

Significant differences among treatments were observed (Table 1). Immersion in mineral oil at 0.5% and insecticidal soap at 2.0% and 0.5% significantly affected more leaves as compared to the control; rinsing of cuttings after immersion in mineral oil did not decrease phytotoxicity (Figure 1). Although immersion in 0.5% insecticidal soap caused some phytotoxicity, as manifested by slight burning of the leaf margins, this was considered to be acceptable. A visual assessment of the cuttings by a local commercial grower confirmed that the lowest levels of phytotoxicity (between 1 and 2 leaves affected) caused by the treatments were acceptable, with no negative aesthetic impact likely by the end of the production period.

**Figure 2:**
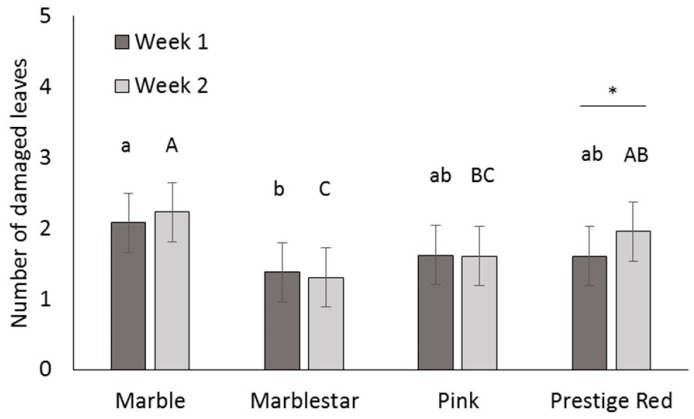
Phytotoxicity (mean ± SE of damaged leaves per cutting) of cutting dips on four greenhouse poinsettia varieties, one and two weeks after dipping. Different lowercase letters indicate significant differences among varieties one week after dipping, different uppercase letters indicate significant differences among varieties at week 2. Asterisk indicates a significant difference between week 1 and 2. (α = 0.05).

Marble and Prestige Red were the most susceptible varieties, Marblestar was the least susceptible variety and susceptibility of Pink was intermediate (Table 1) (Figure 2). The variety by week interaction was also significant (Table 1); within variety, there were no significant differences between weeks, except for Prestige Red, where the number of damaged leaves increased in week 2.

### 3.2. Compatibility of Entomopathogens with Products in Combination Treatments

The viability of *I. fumosorosea* and *B. bassiana* was not adversely affected over 4 h when combined with insecticidal soap, AquaGro, or mineral oil (90% ± 2%). Viability of *S. feltiae* exceeded 90% at all time points.

### 3.3. Efficacy of Immersion in Reduced Risk Materials

**Figure 3:**
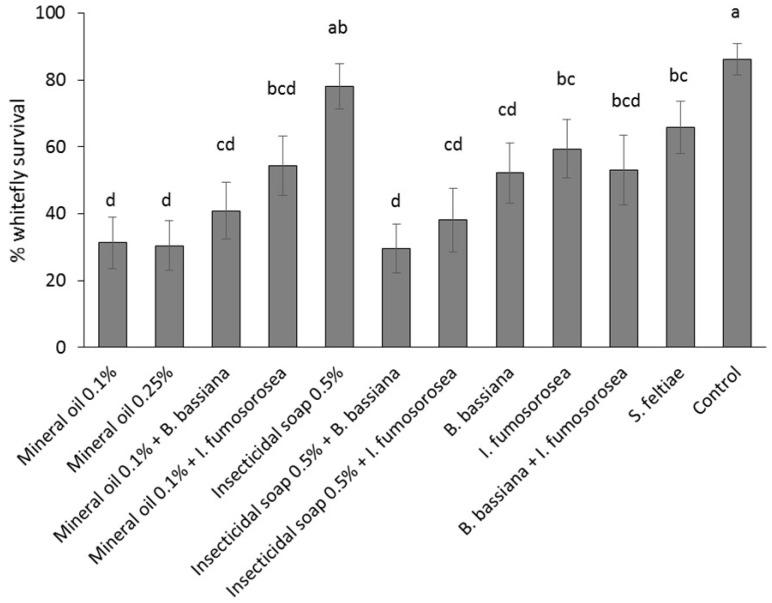
Efficacy (mean percent survival 2 weeks post-treatment ± SE) of reduced risk and biopesticide products against *Bemisia tabaci* (eggs, first and second instar) on greenhouse poinsettia cuttings. Different letters indicate significant differences in efficacy among products (α = 0.05).

Survival of whiteflies varied significantly among treatments (*F* = 5.01, df = 11, *p* = 0.0054) (Figure 3). The control treatment had the highest survival of whiteflies and was not significantly different from the insecticidal soap. Survival was reduced compared to the control in the *S. feltiae* and *I. fumosorosea* treatments. The remaining treatments were all not significantly different from the most efficacious treatments. In general, the entomopathogenic fungi, *B. bassiana* and *I. fumosorosea*, were moderately effective alone or combined. Mineral oil was most effective at both concentrations and combining mineral oil with *B. bassiana* or *I. fumosorosea* did not improve treatment efficacy. Combining insecticidal soap with either of the entomopathogens significantly increased efficacy.

**Figure 4:**
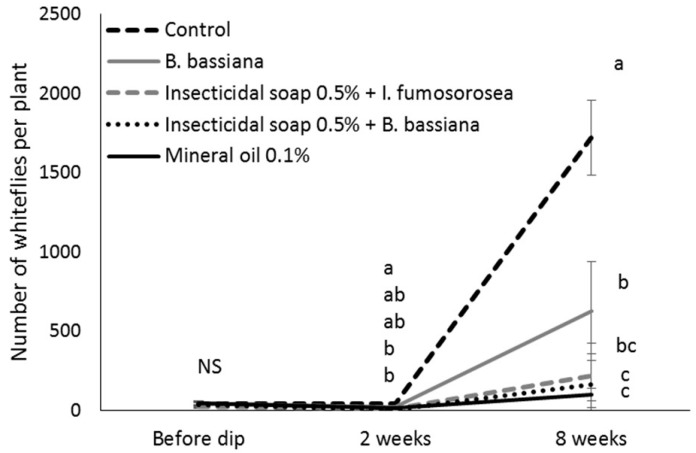
Number (untransformed means ± SE) of *Bemisia tabaci* on greenhouse poinsettia cuttings before and after dipping in selected reduced risk and biopesticide products. Different letters indicate significant differences among time points and treatments (α = 0.05).

All plants treated as cuttings had significantly fewer whiteflies on them than the control plants eight weeks after they were treated (treatment *F* = 7.44, df = 4, *p* = 0.002; weeks *F* = 72.47, df = 2, *p* < 0.0001; treatment*weeks *F* = 11.16, df = 8, *p* < 0.001) (Figure 4). Fewest whiteflies were observed on plants that were dipped as cuttings in mineral oil 0.1%, insecticidal soap + *B. bassiana* and insecticidal soap + *I. fumosorosea*. The plants dipped as cuttings in *B. bassiana* had higher numbers of whiteflies compared to the mineral oil and the insecticidal soap + *B. bassiana* treatments.

Based on these results, the most effective and recommended treatments are mineral oil 0.1% (v/v) and insecticidal soap (0.5% v/v) + *B. bassiana*. Whiteflies increased in all dip treatments, so it is assumed that the observed effects on population development were due to the lower starting populations on the treated plants, rather than there being any residual activity from the dips. No visual effects of treatments on plant growth were observed. For example, the treatments did not affect the number of lateral shoots on the poinsettia plants or plant height.

### 3.4. Compatibility of Dip Treatments with Parasitoids

**Figure 5:**
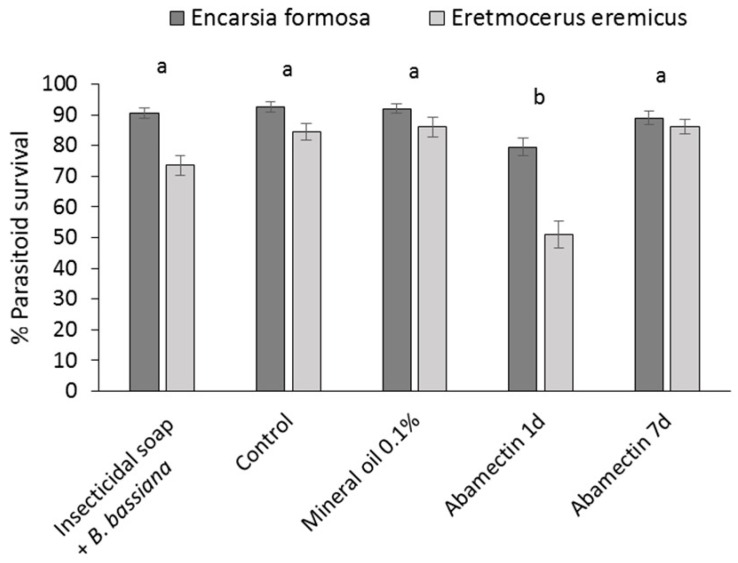
Effect of residues of selected reduced risk and biopesticide products and a pesticide control (Abamectin) on survival of whitefly parasitoids *Encarsia formosa* and *Eretmocerus eremicus* (mean ± SE). Different letters indicate significant differences among products (α = 0.05). Survival of *E. eremicus* was always significantly lower than *E. formosa* (α = 0.05).

Significant effects of dip treatments on parasitoid survival were observed (*F* = 9.56, df = 4, *p* = 0.0021) (Figure 5). One-day-old residues of Abamectin were more harmful to both *E. formosa* and *E. eremicus* than the rest of the dip treatments. Effects were different for the two parasitoid species (species *F* = 21.49, df = 1, *p* = 0.0009). *Eretmocerus eremicus* was always more susceptible to the dip treatments than *E. formosa*. The treatment by species interaction was not significant (*F* = 1.45, df = 4, *p* = 0.2895). Parasitoid survival was consistently above 70%, except for one-day-old residues of Abamectin, where only 51% of *E. eremicus* survived. Therefore, according to the IOBC standards for pesticide compatibility, the treatments may generally be considered harmless, although one-day old residues of Abamectin are considered slightly harmful for *E. eremicus*. Based on these results, the best performing treatments, mineral oil or insecticidal soap + *B. bassiana* treatments, are considered highly compatible with both parasitoid species.

## 4. Discussion

To ensure greater sustainability in poinsettia production, new methods of whitefly control are required that can be applied to cuttings to prevent populations developing beyond the control ‘capacity’ of the parasitoids used, and to ensure that effective biological control systems can be maintained through the crop production cycle. Results of this study showed that dipping cuttings in reduced-risk pesticides, or combinations with microbial biopesticides, significantly reduced *B. tabaci* populations on infested poinsettia cuttings. Phytotoxicity risks of the most efficacious treatments were acceptable, and dip residues on the cuttings had little effect on the survival of commonly used parasitoids (*E. formosa* and *E. eremicus*) and are considered highly compatible.

Mineral oil at 0.1% and the combination of insecticidal soap + *B. bassiana* were the most effective treatments tested. The results obtained are comparable to those obtained in other studies of *B. tabaci* on poinsettia: Petroleum oil products (Certis spray oil and Tri-Tek) applied as a foliar spray or as leaf dip caused high mortality of *B. tabaci* eggs, nymphs and adults, treatments of *B. bassiana* or insecticidal soap (Savona) were moderately effective, and a tank-mix of both Tri-Tek and *B. bassiana* also caused high mortality of *B. tabaci* eggs and nymphs [19,23,28]. Petroleum oil products kill pests by anoxia (suffocation), or through direct toxic effects [29]. In the current study, combining mineral oil with either *B. bassiana* or *I. fumosorosea* did not increase whitefly mortality, contrary to Cuthbertson and Collins [28]. Presumably different application methods (spray vs. dip) or the high efficacy of petroleum oil as a single treatment can explain this difference. In contrast, combining insecticidal soap with the fungal entomopathogens increased overall treatment efficacy. It is possible that removal of cuticular waxes by insecticidal soap made it easier for fungi to infect whitefly.

It is important to note that for dips, the rates for mineral oil and insecticidal soap had to be decreased from the recommended spray rates on the product label to avoid phytotoxicity. The results illustrate the importance of testing product concentrations for a specific application method like dips, rather than assuming that the recommended spray concentrations will be appropriate for all use practices. Recommended label rates for spray application of mineral oil and insecticidal soap negatively affected half of the leaves on each cutting. Although some phytotoxic effects were observed even at the reduced rates tested, visual assessment of the affected cuttings by a grower confirmed that the level of damage was commercially acceptable and that no long-term aesthetic impact was anticipated. The results and recommendations from this study may be extended to other poinsettia varieties and other crops that are started from cuttings. Still, care should be taken to do phytotoxicity tests to confirm that the rates and products that performed best in the current study are suitable. The phytotoxicity experiment showed differences in susceptibility among four popular poinsettia varieties. In addition, a rinse in clean water [11] after a mineral oil dip did not reduce phytotoxicity of these treatments.

The least effective dip treatments were insecticidal soap, *I. fumosorosea* and *S. feltiae*. It is possible that a higher rate of insecticidal soap would be more effective as a dip treatment because foliar sprays of insecticidal soap significantly reduce *B. tabaci* populations (e.g., [30]); however, higher rates in this study caused unacceptably high phytotoxicity on all poinsettia varieties. The combination treatment of the low rate of insecticidal soap and *B. bassiana* caused very high whitefly mortality and should therefore be preferred over the use of insecticidal soap alone.

The rate used for *S. feltiae* was based on the label spray rate for heavy thrips infestations. Cuthbertson et al. obtained higher (ca. 40%) mortality of second instar *B. tabaci* when infested plants were sprayed with *S. feltiae* to run-off at the same rate [22]. At the time of dipping in the current study, cuttings were infested with a mix of *B. tabaci* eggs, first and second instars. The egg stage is considered resistant to nematode infection due to the absence of openings through which the infective juveniles can enter [31], which probably contributed to the overall lower whitefly mortality. In addition, although the nematode suspension was agitated during the dip and a surfactant was used, it is possible that dipping is not the best application method to facilitate nematode infection.

Eight weeks after the dip treatment, surviving whitefly populations had increased, indicating that mineral oil, insecticidal soap and entomopathogens did not have a long-lasting residual effect. This underlines that the most important function of dips is to bring whitefly numbers down to manageable levels at the beginning of the crop production cycle and that follow-up with other control methods, such as biological control has a greater chance of success. This study confirmed that, according to the IOBC standards for pesticide compatibility [27], both mineral oil and insecticidal soap + *B. bassiana* treatments were highly compatible with the whitefly natural enemies *E. formosa* and *E. eremicus*.

The results and recommendations of this study will be validated in long-term experiments that will compare whitefly populations on poinsettia plants grown from dipped and undipped cuttings followed by releases of biological control agents in research and commercial greenhouse settings over the course of a full poinsettia production cycle. It is predicted that, by mitigating *Bemisia* early in the production cycle, populations can be effectively managed thereafter using biological control agents and subsequent pesticide treatments are not required. This will have a positive impact by eliminating pesticide inputs, thereby negating human and environmental risks posed by their use. Furthermore, by emphasizing the use of reduced risk/biological materials, *Bemisia* resistance can also be overcome. The study by Frewin et al. clearly demonstrated the positive benefits of using biological as opposed to chemical control strategies to manage *B. tabaci*, particularly the Mediterranean or Q biotype, which is highly resistant [4].

Future studies will also include a cost-benefit analysis of the dipping strategy. Use of early, preventative strategies are likely to be more economical than curative biological or chemical control programs. Large numbers of cuttings can be rapidly treated by dipping, using relatively small amounts of control products. A highly effective dip will mean that few additional control measures will be required for the rest of the growing season; releases of parasitoids, which are relatively inexpensive, can be made as required. It is possible that the frequency of these releases could be reduced if the dips are highly effective, further reducing the cost of production. Finally, by focusing on early interventions using biopesticide products with minimal restricted entry intervals (REIs), worker access to the crop can be maintained at all times.

Besides pests, the presence of pesticide residues on cuttings due to a heavy reliance on chemical pesticides by propagators is suspected to cause failure of biocontrol programs. Many pesticides are incompatible with beneficial insects through direct toxicity or sub-lethal effects [9]. Preliminary trials found significant (up to 50%) mortality of *E. formosa* and *E. eremicus* on poinsettia cuttings received from a commercial propagator, compared to pesticide residue-free cuttings [32]. Further experiments should be done to determine which pesticides cause this mortality, an assessment of the collective effects of ’sub-lethal’ residues from multiple products on the leaves, how long the residues remain toxic to biocontrol agents, and if there are any methods to accelerate pesticide residue degradation.

Lastly, one of the most cited risks of dip treatments is the transfer and spread of diseases, e.g., the highly infective *Pectobacterium carotovora*, by immersing all cuttings in the same dip suspension. This is a valid concern, and is currently being addressed by quantifying the risks of disease transfer associated with the dipping method, and ways in which these risks can be mitigated, e.g., through the use of biofungicides combined with sanitation practices (the frequency with which dip suspensions are changed) to prevent the potential build-up of inoculum to infectious levels.

## 5. Conclusions

Cutting dips are a promising technology for growers to reduce the risk caused by the introduction of pests on propagative plant material. This research investigated how cutting dips can be applied to control *B. tabaci* on poinsettia cuttings. Although the application rates of mineral oil and insecticidal soap used in the dips are lower than conventional foliar spray rates (to prevent phytotoxicity in poinsettia cuttings), the efficacy of the dips against *B. tabaci* was still high, especially for mineral oil at 0.1% and the combination treatment of insecticidal soap (0.5%) and *B. bassiana* (1.25 g/L). Any residual whitefly populations can be effectively managed later in the production cycle using biological control because dip residues were highly compatible with the commonly used whitefly parasitoids *E. formosa* and *E. eremicus*. The results of this study also lay the foundation for research on the efficacy of biopesticide dips against other important pests of greenhouse ornamentals that are found on cuttings, e.g., western flower thrips (*Frankliniella occidentalis*) and two spotted spider mites (*Tetranychus urticae*).

## Figures and Tables

**Table 1:** ANOVA results of phytotoxicity of reduced risk and biopesticide products applied as cutting dips on four greenhouse poinsettia varieties, one and two weeks after dipping (α = 0.05).

Effect	df	*F*-Value	*p*-Value
Treatment	10	2.17	0.0281
Variety	3	3.46	0.0200
Treatment*variety	30	0.48	0.9879
Week	1	3.37	0.0668
Treatment*week	10	0.66	0.7638
Variety*week	3	3.68	0.0118
Treatment*variety*week	30	0.43	0.9972
